# Phase Transformation Behavior, Mechanical Properties Under Thermal Stress, and Slag-Induced Erosion of 2–4 mol% CeO_2_-Doped CaO-Stabilized Zirconia

**DOI:** 10.3390/ma18010064

**Published:** 2024-12-27

**Authors:** Janghoon Kim, Hwanho Jeon, Kanghee Jo, Hwanseok Lee, Heesoo Lee

**Affiliations:** 1 School of Materials Science and Engineering, Pusan National University, Busan 46241, Republic of Korea; jhoon_k@posco.com (J.K.); jokanghee@pusan.ac.kr (K.J.); 2 POSCO Technical Research Laboratory, POSCO, Gwangyang-si 57807, Republic of Korea; 3 Graduate School of Convergence Science, Pusan National University, Busan 46241, Republic of Korea; hwanhoj@naver.com; 4 GAONE Corp., 3, Godeung-ro, Sujeong-gu, Seongnam-si 13105, Republic of Korea; 5 LiB Recycling Research Center, POSCO Holdings, Pohang-si 37859, Republic of Korea; hwanseok@posco-inc.com

**Keywords:** partially stabilized zirconia, phase stabilization, phase transition, slag-induced erosion, mechanical properties, erosion resistance

## Abstract

We investigated the phase transitions, mechanical properties, and chemical durability of a composition of 9 mol% CaO-stabilized zirconia (9CSZ) doped with 2–4 mol% CeO_2_ under thermal stress against molten slag. The monoclinic phase fraction of 9CSZ was 7.14% at room temperature, and CSZ doped with 2–4 mol% CeO_2_ showed a slightly lower value of 5.55–3.72%, with only a minor difference between them. The microstructure of 9CSZ doped with 2–3 mol% CeO_2_ was similar to that of undoped 9CSZ, whereas the microstructure of 9CSZ doped with 4 mol% CeO_2_ exhibited noticeable grain refinement. The mechanical properties of CSZ at room temperature tended to improve as the CeO_2_ doping concentration increased. The Vickers hardness increased from 1088.4 HV to 1497.6 HV when the CeO_2_ doping amount was 4 mol%, and the specific wear amount decreased from 1.5941 to 1.1320 × 10^5^ mm^3^/Nm. This tendency remained similar even after applying thermal stress. The monoclinic phase fraction of 9CSZ increased from 7.14% to 67.71% after the erosion experiment with the CaF₂-based slag. CeO_2_-doped CSZ had a lower monoclinic phase fraction than CSZ after the erosion experiment, but as CeO_2_ content increased from 2 to 4 mol%, the fraction rose to 4.07%, 30.85%, and 77.11%.

## 1. Introduction

Zirconia (ZrO_2_) has been widely studied due to its excellent mechanical, thermal, and chemical properties, even under extremely harsh conditions. Notably, zirconia exhibits high toughness on par with metals, despite being a ceramic material, which makes it an ideal candidate for a wide range of material development applications, both structural and functional [1,2,3,4]. Molten slag in steelmaking processes is a highly basic and alkaline substance that causes significant chemical erosion of refractory materials. Therefore, zirconia (ZrO_2_), with high mechanical properties and chemical durability under extreme conditions, is used as the refractory material in areas that come into contact with molten slag. Zirconia can exhibit significant changes in crystal structure and mechanical properties depending on the temperature. The monoclinic phase is stable below 1170 °C, the tetragonal phase is stable between 1170 °C and 2370 °C, and the cubic phase is stable above 2370 °C. The transition from the tetragonal to the monoclinic phase involves volume expansion, which causes cracks and grain boundary fractures, leading to a rapid decrease in strength [5,6,7]. Therefore, various research projects have been conducted to improve the mechanical and thermal properties of zirconia used in high-temperature environments by using compounds such as Y_2_O_3_, MgO, Al_2_O_3_, Bi2O_3_, and CaO as phase stabilizers [8,9]. Doping with these stabilizers reduces the transformation of the tetragonal phase, resulting in a structure with lower residual stress within the lattice [10,11]. The type and concentration of stabilizers determine whether zirconia becomes partially stabilized zirconia (PSZ) or fully stabilized zirconia (FSZ) at high temperatures. PSZ and FSZ absorb the stress generated when cracks occur by transforming into the monoclinic phase, which enhances mechanical properties. Therefore, the type and amount of stabilizers affect the phase stabilization of zirconia [12,13,14]. CaO-doped zirconia exhibits superior mechanical properties under high-temperature conditions, such as higher hardness and better high-temperature oxidation resistance, compared to zirconia doped with other stabilizers. Due to these properties and relatively low cost, CaO-doped zirconia is primarily used in structural ceramics like refractories [15,16,17]. CaO-PSZ demonstrates outstanding mechanical and thermal properties, but prolonged use at temperatures exceeding 1500 °C can cause destabilization due to the instability of Ca. Therefore, research is necessary to prevent the destabilization of CaO-PSZ and to improve its mechanical and thermal properties at high temperatures.

Previous research confirmed the trend in the mechanical properties of CaO-PSZ with an increase in CaO doping from 5 to 10 mol%, identifying that 9 mol% CSZ exhibited the most superior mechanical properties [18]. K.H. Heussner demonstrated that doping ZrO_2_ with CeO_2_ in nitrogen or oxygen atmospheres can enhance its mechanical strength [19]. Y. Nigara confirmed that doping CSZ with CeO_2_ improves oxygen permeability [20]. However, studies have not yet explored the changes in mechanical and chemical properties resulting from the additional doping of CeO_2_ in zirconia that has been enhanced through CaO doping.

To further improve the properties and chemical stability of CSZ, we doped 9 mol% CSZ with CeO_2_, which is chemically more stable than CaO and can substitute larger Ce^4+^ ions (97 pm) for Zr^4+^ ions (84 pm) in the lattice, investigating the resulting changes in properties and chemical stability against molten slag.

## 2. Materials and Methods

The 9 mol% CSZ was synthesized using ZrO_2_ (99%, Daejung Chemicals & Metals Co., Siheung-si, Republic of Korea) and CaO (98%, Junsei Chemical Co., Tokyo, Japan) as starting materials through a solid-state method. The synthesized 9CSZ was then doped with CeO_2_ (99.9%, Junsei Chemical Co., Tokyo, Japan) according to the ratios specified in Table 1. The mixture was ball-milled with zirconia balls and ethanol for 24 h, dried at 90 °C for 24 h, and then calcined at 1200 °C with a heating rate of 5 °C per minute for 2 h.

The test specimens were prepared by uniaxially pressing the synthesized powder at a pressure of 1 ton/cm^3^ to form cylindrical (20 mm diameter) and rod-shaped (5 mm width, 35 mm length) bodies. These formed bodies were then sintered in a box-type furnace, with a heating rate of 5 °C per minute, at 1600 °C for 6 h, followed by furnace cooling. The thermal shock test for the specimens involved heating the sintered samples in an air atmosphere to 1300 °C at a rate of 5 °C per minute, holding them at that temperature for 5 min, and then allowing them to cool naturally to 900 °C. This cycle was repeated 40 times to apply thermal shock.

Erosion tests were performed to evaluate the chemical stability of each specimen in the slag. Five grams of CSZ powder was pressed into pellets using a cylindrical mold with a diameter of 15 mm under a pressure of 1 ton/m^2^. The formed CSZ specimens were placed in a graphite crucible, and 500 g of slag, as specified in Table 2, was added. The crucible was then maintained at 1550 °C for 3 h and subsequently cooled in the furnace. Since the slag dissolves in Al_2_O_3_, SiO_2_, and MgO, a graphite crucible, which does not react with the slag, was used for the reactivity test. After cooling was completed following the melting process, the crucible was broken to retrieve the CSZ specimens from within the slag. The inner parts of the specimens, which were free from slag contamination, were then crushed and subjected to XRD analysis.

To evaluate the chemical stability of each specimen, we conducted reactivity tests with slag. The pellet-shaped specimens were placed in a graphite crucible, and the slag specified in Table 2 was added. The samples were then maintained at 1550 °C for 3 h, followed by furnace cooling. Since the slag has solubility in Al_2_O_3_, SiO_2_, and MgO, a graphite crucible, which does not react with the slag, was used for the reactivity tests.

A crystal structure analysis was performed using X-ray diffraction (XRD, Rigaku, Ultima-IV, Japan) with measurements taken from 20° to 80° at a step size of 0.02°/2θ and a scanning speed of 2°/min. The XRD peaks and phase fractions were analyzed using the Rietveld refinement method with Highscore Plus software (version 3.0c) with a reference pattern for monoclinic ZrO_2_ (m-ZrO_2_, ICSD 98-006-0900) and tetragonal ZrO_2_ (t-ZrO_2_, ICSD 98-007-0014). And the monoclinic phase fraction was double-checked for integrated intensity by using ISO 5803 [21].

(1) $$X=\frac{I(\bar{1}11{)}_{m}+I(111{)}_{m}}{I(\bar{1}11{)}_{m}+I(111{)}_{m}+I(101{)}_{t}}$$

(2) $$X=\frac{I(\bar{1}11{)}_{m}+I(111{)}_{m}}{I(\bar{1}11{)}_{m}+I(111{)}_{m}+I_{ttc}}$$

Equation (1) is for a two-phase system (monoclinic and tetragonal phases), and Equation (2) is for a multi-phase system (a mixture of monoclinic, tetragonal, and cubic). *X* is the integrated intensity ratio, where $I(\bar{1}11{)}_{m}$ and $I(111{)}_{m}$ refer to the integral intensity of the monoclinic X-ray diffraction pattern. $I(101{)}_{t}$ refers to the integral intensity from the (101) plane of the tetragonal and $I_{ttc}$ is the total integrated intensity of the tetragonal phase (101) and cubic phase (111) reflections. The volume fraction of the monoclinic phase was calculated using Equation (3):

(3) $$f_{m}=\frac{PX}{1+\left(P-1\right)X}$$

$f_{m}$ is the volume fraction of the monoclinic phase and *P* is the intensity factor. In the monoclinic-tetragonal ZrO_2_ system, *P* = 1.219 was used, while in the multiphase system, *P* = 1.265 was used.

We used a high-resolution scanning electron microscope (HR-SEM, SU8230, Hitachi, Tokyo, Japan) to observe the microstructural changes on the specimen surfaces. The mechanical properties were assessed using a micro Vickers hardness tester (Wilson, VH1102, Lake Bluff, IL, USA) under test conditions of 20 N load and a dwell time of 15 s. Each measurement was repeated 10 times, and the Vickers hardness was calculated using the average value. Wear resistance was measured by determining the specific wear rate (m^3^/N) under the conditions of a sliding distance of 1000 m, a load of 9.6 N, a velocity of 0.1 m/s, and an operating type of ball-on-disk. The measurements were conducted in accordance with the ISO 20808 testing method [22]. The flexural strength of each specimen was measured using a universal testing machine from the United States, with a lower support-point distance of 20 mm and a crosshead speed of 0.5 mm/min in a three-point bending test.

## 3. Results and Discussion

**Figure 1:**
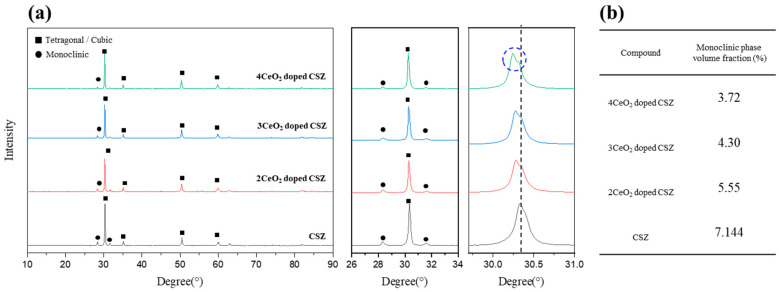
(**a**) XRD diffraction patterns of CeO_2_-doped 9CSZ powder calcinated at 1600 °C (•: monoclinic phase, ◼: tetragonal or cubic phase), (**b**) Phase fraction determined through Rietveld refinement.

The results of the phase analysis after doping CeO_2_ at 2–4 mol% into 9 mol% CSZ are shown in Figure 1. The monoclinic fraction (V_m_) of zirconia was 7.14% for 9CSZ, while under doping conditions of 2–4 mol% CeO_2_, it decreased slightly to 5.55% and 3.72%, respectively, compared to CSZ, without showing any abrupt change. CeO_2_ doping has a lesser effect on improving phase stability at room temperature because the Ce^4+^ ion has the same charge state as the Zr^4+^ ion, resulting in no formation of oxygen vacancies due to charge compensation. Phase stabilization is induced by lattice stress caused by the ionic radius difference between Zr^4+^ (84 pm) and Ce⁴⁺ (97 pm). The doping of Ce^4+^, which has a larger ionic radius than Zr^4+^, leads to lattice contraction, which is observed as a low-angle shift in the XRD pattern. In particular, the peak splitting between 30° and 30.5° observed in 4 mol% CeO₂-doped CSZ suggests the formation of a cubic phase.

**Figure 2:**
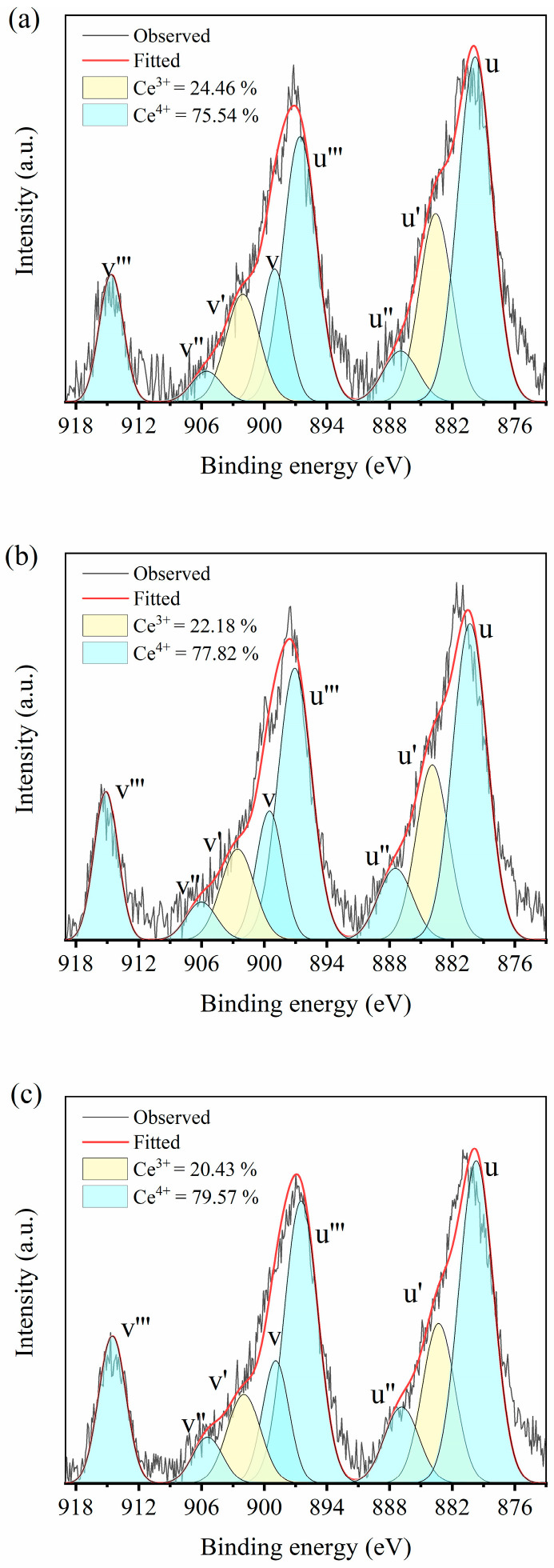
Ce 3d XPS spectrum and deconvoluted cures for Ce^3+^ and Ce^4+^ after background subtraction of (**a**) 2Ce_CSZ, (**b**) 3Ce_CSZ, and (**c**) 4Ce_CSZ.

Figure 2 shows the Ce 3d XPS spectrum of CeO_2_-doped CSZ after wet ball milling and calcination at 1200 °C in an air atmosphere for 2 h. The sub-bands denoted as u′ and v′ in the XPS spectrum corresponded to the initial electron state of 3d_10_4f_1_ of Ce^3+^, while u, u′′, u′′′, v, v′′, and v′′′ represented the 3d_10_4f_0_ state of Ce^4+^ [23]. It is evident that Ce predominantly existed in the form of Ce^4+^ within the zirconia lattice, and with increasing CeO_2_ doping, the proportion of Ce^4+^ increased from 73.98% in 1Ce_CSZ to 79.57% in 4Ce_CSZ. This trend indicates that local lattice tension increased with increased CeO_2_ doping due to the ionic radius differences among Ce^3+^ (114 Å), Ce^4+^ (97 Å), and Zr^4+^ (86 Å). The proportion of Ce^3+^ decreased to alleviate local lattice tension induced by the difference in ionic size [24]. The stabilizing effect of CeO_2_ doping is primarily due to lattice stress caused by the ionic radius difference between Zr^4+^ and Ce^4+^, not due to oxygen vacancy formation by charge compensation of Ce^3+^ [25]. CeO_2_ doping leads to an increase in the tetragonal phase, which can lead to improving mechanical properties by transformation toughening under external mechanical stress [26,27].

**Figure 3:**
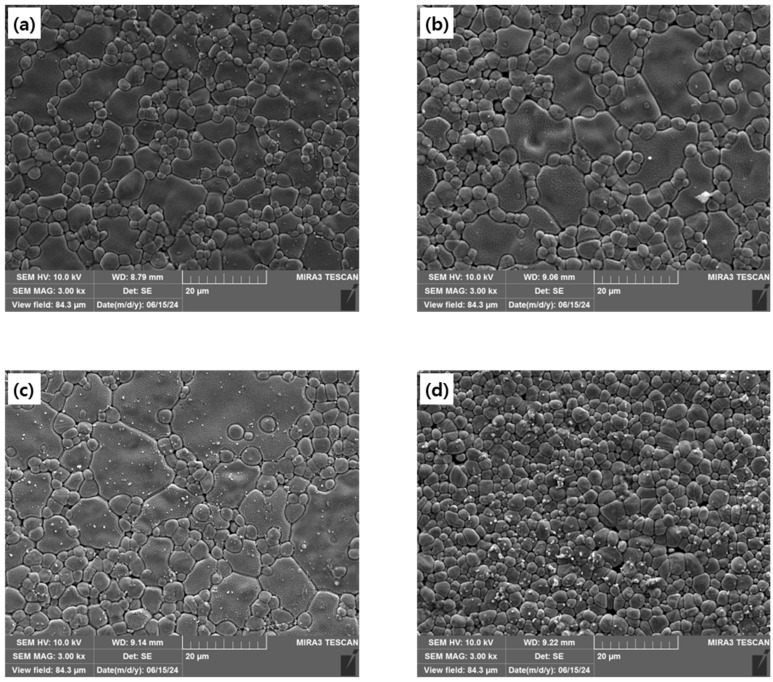
FE-SEM images and average grain sizes of (**a**) 9CSZ, (**b**) 2CeO_2__CSZ, (**c**) 3CeO_2__CSZ, and (**d**) 4CeO_2__CSZ specimens after sintering.

Figure 3 shows the microstructures of each specimen. The grain size of 9CSZ doped with 2–3 mol% CeO_2_ was comparable to that of 9CSZ, exhibiting a mixture of large grains ranging from 11.9 to 18 μm and small grains below 6 μm. In contrast, 9CSZ doped with 4 mol% CeO_2_ showed only small grains below 5.8 μm. The changes in the microstructural distribution in 4 mol% CeO_2_-doped CSZ are thought to occur because the increased amount of CeO₂ doping, which does not sinter well, prevents grain growth during heat treatment.

**Figure 4:**
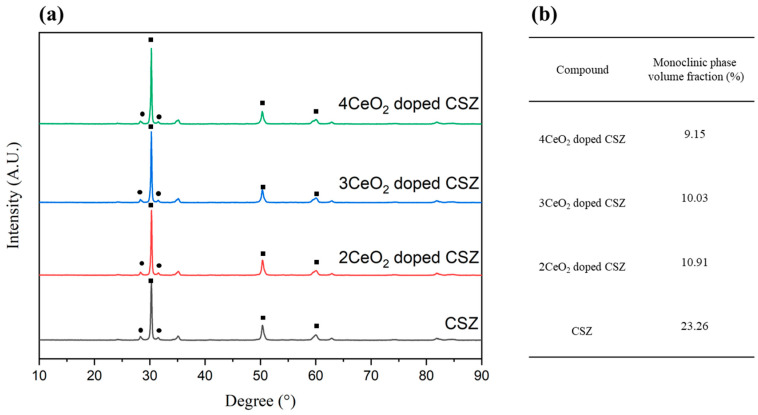
(**a**) XRD diffraction patterns of CeO_2_-doped 9CSZ after thermal shock (△400 °C) (•: monoclinic phase, ◼: tetragonal or cubic phase), (**b**) Phase fraction determined through Rietveld refinement.

The phase analysis results after applying thermal stress to 2–4 mol% CeO_2_-doped CSZ are shown in Figure 4. The monoclinic phase fraction (V_m_) of zirconia was significantly increased from 7.14% to 23.26% in the case of 9CSZ, whereas under the 2–4 mol% CeO_2_ doping conditions, it was relatively less changed to 10.91%, 10.03%, and 9.15%, respectively. These results indicate that the phase stability of 9CSZ with CeO_2_ improved under thermal stress. The reduction in oxygen vacancies and the minimization of lattice structure distortion are believed to be caused by the substitution of Ce^4+^, which has the same electric valence state as Zr^4+^ and a similar ionic size.

**Figure 5:**
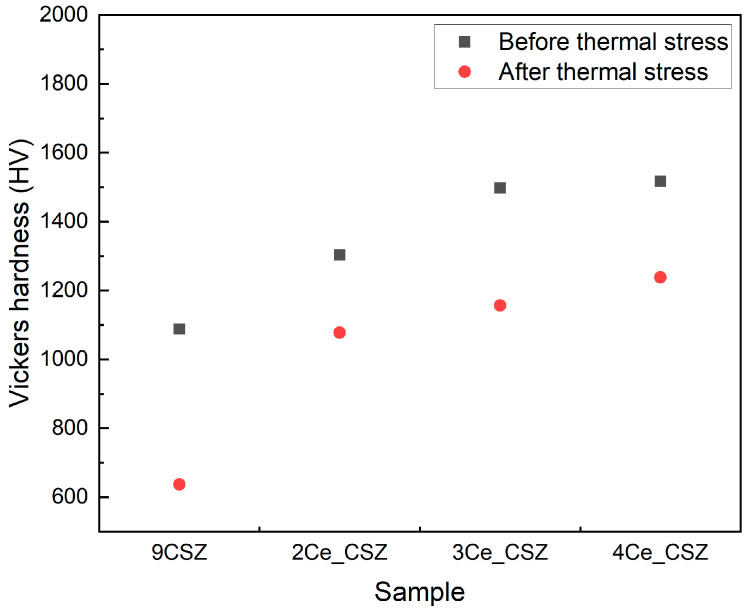
Vickers hardness of CeO_2_-doped CSZ before (black scattered point) and after (red scattered point) post-heat treatment.

The mechanical properties of each specimen were observed after subjecting 2–4 mol% CeO_2_-doped CSZ to thermal shock under Δ400 °C (1300 and 900 °C) conditions. Figure 5 shows the changes in Vickers hardness before and after thermal shock, indicating that the Vickers hardness gradually increased with an increasing amount of CeO_2_ doping. The Vickers hardness of all specimens decreased after applying thermal shock. Specifically, 2–4 mol% CeO_2_-doped CSZ showed a decrease of 20.3% (1254.9→1000.1 HV), 17.5% (1303.4→1077.9 HV), and 22.8% (1497.6→1156.7 HV), respectively. In contrast, 9CSZ exhibited a relatively larger decrease of approximately 41.5% (1088.4→637.0 HV).

**Figure 6:**
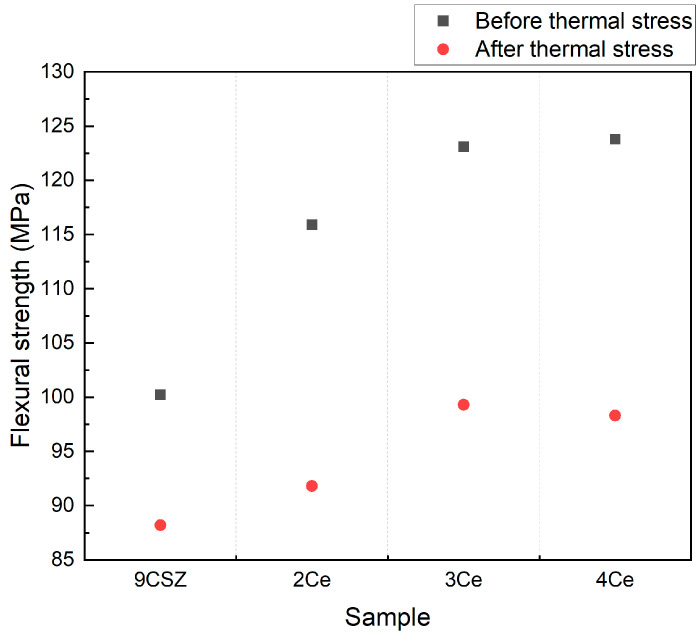
Flexural strength of CeO_2_-doped CSZ before(black scattered point) and after (red scattered point) post-heat treatment.

The flexural strength before and after thermal shock is shown in Figure 6. The flexural strength gradually increased with an increase in CeO_2_ doping content, and the flexural strength of all specimens decreased after thermal shock. Specifically, the flexural strength of 9 mol% CSZ before the thermal shock test was 100.23 MPa, while the flexural strength of 2–4 mol% CeO_2_-doped CSZ was 115.9 MPa, 123.1 MPa, and 123.8 MPa, respectively. After thermal shock, the flexural strength of 9 mol% CSZ was 88.2 MPa, and the flexural strength of 2–4 mol% CeO_2_-doped CSZ was 91.38 MPa, 99.3 MPa, and 98.3 MPa, respectively. The decrease in flexural strength of CeO_2_-doped CSZ after the thermal shock was greater compared to 9 mol% CSZ, but it still exhibited higher values than 9 mol% CSZ even after the thermal shock. The increase in flexural strength for 4 mol% CeO_2_-doped CSZ was smaller compared to 3 mol%-doped CSZ, which was consistent with the trends observed in the Vickers hardness results.

**Figure 7:**
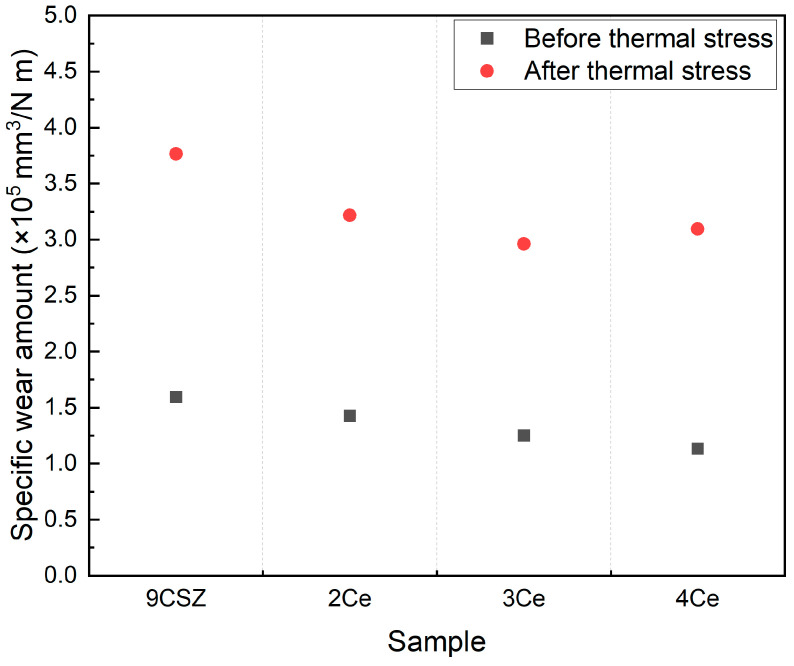
Specific wear amount of CeO_2_-doped CSZ before (black scattered point) and after (red scattered point) post-heat treatment.

The results of the wear resistance evaluation of the specimens before and after thermal shock are shown in Figure 7. The wear amount of the specimens decreased with increasing CeO_2_ doping levels, and all specimens exhibited an increase in wear amount after thermal shock, which was consistent with the trend observed in Vickers hardness. The specific wear amount of 9CSZ increased by 136.2% (1.5941→3.7650 × 10^5^ mm^3^/Nm) before and after thermal stress. CeO_2_-doped 9CSZ exhibited specific wear amount changes of 125.4% (1.4265→3.2160 × 10^5^ mm^3^/Nm), 136.7% (1.2512→2.9610 × 10^5^ mm^3^/Nm), and 173.4% (1.1320→3.0951 × 10^5^ mm^3^/Nm) before and after thermal stress as the CeO_2_ doping level increased from 2 to 4 mol%. As the CeO_2_ doping level increased, the increase in the wear amount before and after thermal stress also increased, with a particularly sharp change observed in the 4 mol% CeO_2_-doped CSZ. The sharp increase in the wear amount before and after thermal stress in the 4 mol% CeO_2_-doped CSZ is believed to be related to the formation of the cubic phase, as indicated by XRD analysis.

The results of Figure 5, Figure 6 and Figure 7 show that doping 9CSZ with CeO_2_ can further enhance the mechanical properties of CSZ. However, it was identified that doping with more than 4 mol% CeO_2_ leads to a decrease in the property stability of CSZ after thermal stress.

**Figure 8:**
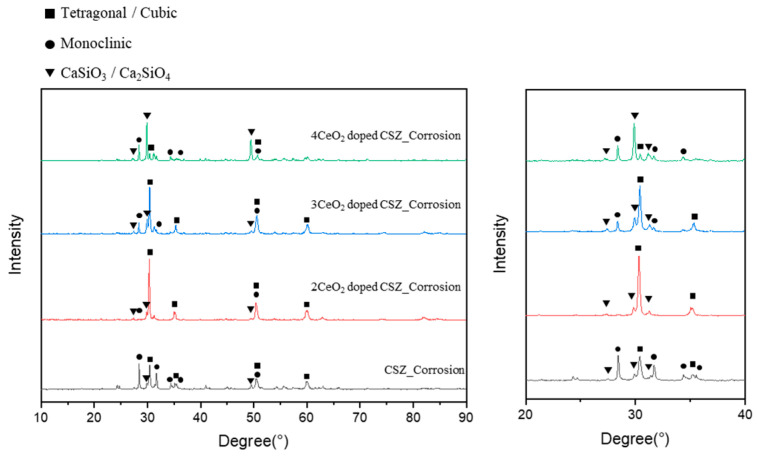
X-ray diffraction patterns of CeO_2_-doped 9CSZ after erosion experiment in slag.

Figure 8 shows the phase transition of the CeO_2_-doped CSZ after an erosion test with a highly basic CaF_2_-based slag to examine the effect of CeO_2_ doping on the erosion resistance of CSZ. The monoclinic phase fraction of 9CSZ increased significantly from 7.144% to 67.71% after the erosion test, while 2Ce_CSZ and 3 Ce_CSZ showed a relatively lower monoclinic phase fraction of 4.07% and 30.85%. The 4Ce_CSZ showed a higher monoclinic phase faction of 77.12%. As the CeO_2_ content increased, the monoclinic phase fraction also tended to increase, with the 2 mol% CeO_2_ doping condition showing the lowest monoclinic phase fraction.

The higher phase stability of CeO_2_-doped CSZ after chemical erosion with CaF_2_-based slag is considered to be due to the very low solubility of CeO_2_ in the slag, unlike CaO, which prevents the dopant from chemically dissolving into the slag at high temperatures. However, excessive CeO_2_ doping can lead to structural instability in CSZ, so it is important to investigate the optimal doping concentration.

## 4. Conclusions

We doped 9 mol% CaO-stabilized ZrO_2_ with 2–4 mol% CeO_2_ to investigate the mechanical and chemical changes due to CeO_2_ doping. The mechanical property changes under thermal stress and the behavior of the CSZ composition and phase changes after an erosion test in CaF_2_-based slag were then observed. The monoclinic phase fraction (V_m_) of zirconia was 7.14% for 9CSZ, while under doping conditions of 2–4 mol% CeO_2_, it decreased slightly to 5.55% and 3.72%, respectively, compared to CSZ, without showing any abrupt change. The Ce^4+^ ion had the same charge state as the Zr^4+^ ion, resulting in no formation of oxygen vacancies due to charge compensation. Phase stabilization was induced solely by lattice stress caused by the difference in the ionic radii between Zr^4+^ (84 pm) and Ce^4+^ (97 pm). Therefore, doping with a small amount of CeO_2_ is presumed to have a lesser effect on improving phase stability at room temperature. In particular, the peak splitting between 30° and 30.5° observed in 4 mol% CeO₂-doped CSZ suggested the formation of a cubic phase. The grain size of 9CSZ doped with 2–3 mol% CeO_2_ was comparable to that of 9CSZ, while 9CSZ doped with 4 mol% CeO_2_ exhibited finer microstructure. This is presumed to be due to the cubic phase formed at 4 mol% doping.

The Vickers hardness increased from 1088.4 HV to 1497.6 HV as the CeO_2_ doping amount in 9CSZ increased. Even after applying a thermal shock of △400 °C (1300→900 °C), the CeO_2_-doped 9CSZ showed relatively higher values, increasing from 637.0 HV to 1156.7 HV, compared to 9CSZ. The specific wear amount before thermal stress decreased to 1.4265 × 10^5^ mm^3^/Nm, 1.2512 × 10^5^ mm^3^/Nm, and 1.1320 × 10^5^ mm^3^/Nm with the addition of 2–4 mol% CeO_2_ doping, compared to the specific wear amount of CSZ, which was 1.5941 × 10^5^ mm^3^/Nm. The specific wear amount of 9CSZ increased by 136.2% (1.5941→3.7650 × 10^5^ mm^3^/Nm) before and after thermal stress. CeO_2_-doped 9CSZ exhibited specific wear amount changes of 125.4% (1.4265→3.2160 × 10^5^ mm^3^/Nm), 136.7% (1.2512→2.9610 × 10^5^ mm^3^/Nm), and 173.4%(1.1320→3.0951 × 10^5^ mm^3^/Nm) before and after thermal stress as the CeO_2_ doping level increased from 2 to 4 mol%. As the level of CeO_2_ doping increased, the increase in wear amount before and after thermal stress also increased, with a particularly sharp change observed in CSZ doped with 4 mol% CeO_2_. The sharp increase in wear amount before and after thermal stress in CSZ doped with 4 mol% CeO_2_ is believed to be related to the formation of the cubic phase generated in 4 mol% CeO_2_-doped CSZ.

Doping CeO_2_ into 9CSZ resulted in a lower formation of the monoclinic phase compared to 9CSZ, even under the chemical reaction conditions with CaF_2_-based slag at 1550 °C. These results suggested that CeO_2_ doping in CSZ can enhance phase stability under high temperature and high alkalinity conditions. However, the monoclinic phase fraction was lowest at 2 mol% CeO_2_ doping, and it showed an increasing trend as the doping concentration increased beyond that.

As a result, CeO_2_ doping was found to improve the mechanical properties of 9 mol% CSZ and enhance its stability against CaF_2_-based slag at high temperatures. However, doping with more than 4 mol% CeO_2_ can cause a decrease in mechanical properties under thermal stress conditions of 1300–900 °C due to the formation of the cubic phase. Therefore, it is important to optimize the CeO_2_ doping concentration according to the operating conditions.

## Figures and Tables

**Table 1:** The composition of the 9CSZ specimens with different amounts of CeO_2_.

Compound	Composition	
	9CSZ	CeO_2_ (mol%)
9CSZ	Balance	0
9CSZ_2CeO_2_		2
9CSZ_3CeO_2_		3
9CSZ_4CeO_2_		4

**Table 2:** Chemical composition of slag powder.

Slag Powder	SiO_2_	CaO	Na_2_O	MgO	Al_2_O_3_	F	C
Composition (wt%)	33.30	31.60	11.10	1.44	2.38	9.10	11.08

## Data Availability

The raw data supporting the conclusions of this article will be made available by the authors upon request.
